# Posttransplant *de novo* DSA and NDSA affect GvHD, OS, and DFS after haplo-HSCT in patients without pre-existing HLA antibodies of hematological malignancies

**DOI:** 10.3389/fimmu.2022.1047200

**Published:** 2022-12-01

**Authors:** Lan Wang, Kai Ji, Luyao Chen, Ying Li, Wenjuan Zhu, Xiaoni Yuan, Xiaojing Bao, Xiaojin Wu, Jun He

**Affiliations:** ^1^ HLA Laboratory of Jiangsu Institute of Hematology, The First Affiliated Hospital of Soochow University, Suzhou, Jiangsu, China; ^2^ Department of Hematology, Jiangsu Institute of Hematology, First Affiliated Hospital of Soochow University, Suzhou, Jiangsu, China; ^3^ Collaborative Innovation Center of Hematology, Soochow University, Suzhou, Jiangsu, China

**Keywords:** *de novo* human leukocyte antigen antibody (*de novo* HLA antibody), donor-specific anti-HLA antibody (DSA), acute graft-versus-host disease (aGvHD), haploidentical hematopoietic stem cell transplantation (haplo-HSCT), outcomes

## Abstract

To examine the production time, type, and MFI of post-transplantation *de novo* HLA antibodies, and their effects on haplo-HSCT outcomes, we retrospectively included 116 patients who were negative for pre-existing HLA antibodies. In total, 322 serum samples from pre-transplantation to post-transplantation were dynamically tested by Luminex and single-antigen bead reagents. Patients were divided into: HLA antibody persistently negative group (group 1), the *de novo* HLA antibody transiently positive group (group 2), the *de novo* HLA antibody non-persistently positive group (group 3), and the *de novo* HLA antibody persistently positive group (group 4). Group 4 included DSA+non-DSA (NDSA) (group 4a) and NDSA (group 4b) groups. The detection rate of *de novo* HLA antibodies was 75.9% (88/116). The median MFI for *de novo* HLA antibodies was 2439 (1033-20162). The incidence of II–IV aGvHD was higher in group 2 than in group 1 (52.6% vs 17.9%, P < 0.01); in group 4a than in group 1 (87.5% vs 17.9%, P < 0.001); and in group 4a than in group 4b (87.5% vs 40.0%, P = 0.001). The DFS (37.5% vs 85.7%, P < 0.01) and OS (37.5% vs 85.7%, P < 0.01) of group 4a were lower than those of group 1. The DFS (48.0% vs 85.7%, P < 0.01) and OS (56.0% vs 85.7%, P = 0.03) of group 4b were lower than those of group 1. Multivariate analysis showed that *de novo* HLA antibody being transiently positive (HR: 5.30; 95% CI: 1.71–16.42, P = 0.01) and persistently positive (HR: 5.67; 95% CI: 2.00–16.08, P < 0.01) were both associated with a higher incidence of II–IV aGvHD. Persistently positive *de novo* HLA antibodies were a risk factor for reduced DFS (HR: 6.57; 95% CI: 2.08–20.70, P < 0.01) and OS (HR: 5.51; 95% CI: 1.73–17.53, P < 0.01). DSA and NDSA can be detected since 15 days after haplo-HSCT in patients without pre-existing HLA antibodies, and affect aGvHD, DFS, and OS. Haplo-HSCT patients must be monitored for HLA antibodies changes for appropriate preventive clinical management, and we recommend that 1-month post-transplantation is the best test time point.

## 1 Introduction

Haploidentical hematopoietic stem cell transplantation (haplo-HSCT) is an effective treatment for hematologic malignancy patients (1–4). The 2018 guidelines published by the European Society for Blood and Marrow Transplantation included human leukocyte antigen (HLA) antibody testing as routine for haplo-HSCT patients (5). Donor-specific HLA antibodies (DSA) are defined as HLA antibodies in the serum of patients that target donor HLA antigens, and the donor HLA antigens are mismatched with those of the patients. Previous studies have shown that the presence of pre-existing HLA antibodies, particularly DSA, can mediate transplant failure, delay hematopoietic reconstitution, and result in poor graft function, thereby affecting patient outcomes (6–11). It is worth noting that *de novo* DSA can cause a rejection reaction through antibody-dependent cellular cytotoxicity or complement-dependent cytotoxicity in organ transplantation. Therefore, it is widely reported that DSA can mediate post-transplantation rejection reactions (12–14). In haplo-HSCT, the recipient tested negative for pre-existing HLA antibodies may produce *de novo* HLA antibodies after transplantation when at least one HLA haplotype is mismatched. However, there is no study in China or other countries on the effects of *de novo* HLA antibodies and their dynamic changes on haplo-HSCT outcomes.

In this study, we included patients who underwent haplo-HSCT and were negative for pre-existing HLA antibodies. All recipients underwent high-resolution typing of HLA alleles. All patients underwent at least one post-transplantation HLA antibody test. We adopted single-antigen bead reagents to detect DSA or NDSA and their MFI values and determined the specificity of *de novo* antibodies and their dynamic changes. We also analyzed their effects on hematopoietic reconstitution, acute graft-versus-host disease (aGvHD), recurrence, and survival outcomes in haplo-HSCT patients.

## 2 Materials and methods

### 2.1 Sample collection and test time points

In this study, we included 216 patients with hematologic malignancy who underwent HLA antibody testing before and after haplo-HSCT in the First Affiliated Hospital of Soochow University from 2016 to October 2020. In total, 100 patients were positive for pre-existing HLA antibodies and 116 patients were negative for pre-existing HLA antibodies. These 116 patients were included in this study, while the other 100 HLA antibody positive patients will be reported in another paper. The inclusion criteria include: 1) A hematologic malignancy, including acute myeloid leukemia (AML), acute lymphoblastic leukemia (ALL), myelodysplastic syndrome (MDS), and lymphoma; 2) A negative pre-transplantation HLA antibody test and at least 1 HLA antibody specificity test after transplantation. The post-transplantation test time points include 15 days, 1 month, 3 months, 6 months, and 1 year. A total of 322 serum samples were collected for testing. All enrolled patients were followed up until 30 January 2021.

The studies involving human participants were reviewed and approved by the Institutional Review Board at First Affiliated Hospital of Soochow University. Written informed consent was obtained from the participants and minors’ legal guardian for participation in this study and for the publication of any potentially identifiable images or data included in this article.

### 2.2 Detection of and judgment standards for HLA antibodies

The collected serum samples were first examined by mixed screening of HLA antibodies (MIX). When the results of mixed screening were positive, HLA-I- or II-specific antibody test was performed to determine the type of HLA antibodies (LABScreen@Single Antigen HLA Class I (LS1 A04) and HLA Class II (LS2A01), One Lambda) and the mean fluorescence intensity (MFI). HLA antibody reactivity was determined using the Luminex platform (Luminex, Canoga Park, CA).

The judgment standards for the MFI values of HLA antibodies were determined according to the standards of the HLA Laboratory of First Affiliated Hospital of Soochow University (15). HLA antibodies with an MFI above 1000 were considered positive. MFI was divided into different groups: between 1000 and 2000, between 2000 and 5000, between 5000 and 8000, and over 8000.

### 2.3 Definition of HLA antibody groups

Group 1 was the HLA antibody persistently negative group and included patients who were negative before transplantation and had at least one or two negative post-transplantation test results. Group 2 was the *de novo* HLA antibody transiently positive group: these patients were negative before transplantation, had 1 positive post-transplantation *de novo* HLA antibody test result, and had at least 1 single-antigen bead (SAB) test during follow-up to show that they converted to negative. Group 3 was the *de novo* HLA antibody non-persistently positive group: these patients were negative before transplantation, had 1 positive post-transplantation *de novo* HLA antibody test result, but did not have any HLA antibody test during follow-up to show whether they converted to negative or were persistently positive for HLA antibodies. Group 4 was the *de novo* HLA antibody persistently positive group: *de novo* HLA antibodies were detected in at least two follow-up tests and the last follow-up test result was still positive.

### 2.4 Condition regimen and graft-versus-host disease prophylaxis

All patients underwent myeloablative conditioning. Specifically, 104 patients received the Bu/Cy-based (busulfan/cyclophosphamide) regimen, with MECCNU (250 mg/m^2^) on day -10, Bu (3.2 mg/kg/day) on days −7 to −5, Ara-C (4 g/m^2^/day) on days −9 to −8, and CTX (1.8 g/m^2^/day) on days −4 to −3; 8 patients received TBI/Cy-based (total body irradiation) as the main pretreatment, with MECCNU (250 mg/m^2^) on day -8, Ara-C (4 g/m^2^/day) on days −6 to −5, CTX (1.8 g/m^2^/day) on days −4 to −3, and TBI (with a total dose of 8-8.5 Gy) on days -7 to -6. One patient was treated with a Bu+ATG regimen, one with a Bu+fludarabine (30 mg m^2^/day) regimen, one with a TBI+fludarabine regimen, and one with a TBI+Bu/Cy regimen.

GvHD prevention included continuous cyclosporine A infusion at 3 mg/kg/day over 24 h from day -10 until patients could switch to oral intake; and mycophenolate mofetil oral formulation at 15 mg/kg/12 h from day −9 to day +30, then gradually tapered until day +60; short-term methotrexate on days +1, +3, +6, and +11 at doses of 15, 10, 10, and 10 mg/kg/day, respectively; and anti-thymocyte globulin on days −5 to −2 at 2.5 mg/kg/day.

### 2.5 Observed indicators

Platelet reconstitution was defined as the first of 7 consecutive days of platelet counts >20×10^9^/L without transfusion support. Graft rejection was defined as a failure to engraft neutrophils (absolute neutrophil count (ANC) ≤0.5 × 10^9^/L) by day +28 for three consecutive days and the absence of donor hematopoiesis (6). Grades of aGvHD were assessed according to published consensus criteria (16, 17). Overall survival (OS) was defined as survival between transplantation and the end of follow-up or death due to any cause. Disease-free survival (DFS) was calculated from the day of transplantation until death, relapse, primary graft failure, or the last follow-up. Relapse was determined using standard hematological criteria. Non-relapse mortality (NRM) was defined as death from all causes other than relapse.

### 2.6 Statistical analysis

The nonparametric test (Mann-Whitney test) was employed to compare continuous variables, and the chi-square test (Fisher’s exact test) was utilized to compare categorical variables. Survival analyses were carried out using Kaplan-Meier methods, and curves were compared by the log-rank test using R version 4.0.5 software packages (R Development Core Team, Vienna, Austria). A competitive risk model was applied to calculate the cumulative incidence of GvHD, relapse and NRM. Death was considered a competitive risk for aGvHD, relapse and NRM. Multivariable analyses for aGvHD and relapse were performed using competing risks regression. OS and DFS were determined using the Cox proportional hazards model in SPSS version 25.0 (IBM, Armonk, New York, USA). All test results with two-sided p<0.05 were considered statistically significant.

## 3 Results

### 3.1 *De novo* HLA antibody test result grouping and clinical characteristics of 116 patients

The 116 enrolled patients were classified based on test results into group 1 (n = 28), group 2 (n = 19), group 3 (n = 36), and group 4 (n = 33). There were no significant differences in the clinical characteristics (recipient age, gender, relationship, or diagnosis) of the four groups of patients and their donors (**Table 1**).

**Table 1:** Clinical characteristics of 116 patients with malignant hematological diseases.

	Group 1	Group 2	Group 3	Group 4	P
Total number	28	19	36	33	
Median age, (range), year					
Patient	36.5 (14-58)	31 (16-59)	27.5 (8-66)	33 (13-58)	0.30
Donor	32 (10-58)	42 (17-57)	38.5 (7-58)	39 (13-630)	0.16
Median time from diagnosis to HSCT, (range), month	5.5 (1-16)	5 (1-29)	6 (2-83)	4 (3-63)	0.87
Recipient sex, n (%)					0.47
Man	16 (57.1)	10 (52.6)	25 (69.4)	23 (69.7)	
Female	12 (42.9)	9 (47.4)	11 (30.6)	10 (30.3)	
Disease, n (%)					0.78
AML	8 (28.6)	6 (31.6)	17 (47.2)	14 (42.4)	
ALL	12 (42.9)	9 (47.4)	10 (27.8)	12 (36.4)	
MDS	6 (21.4)	2 (10.5)	6 (16.7)	6 (18.2)	
Lymphoma	2 (7.1)	2 (10.5)	3 (8.3)	1 (3.0)	
Donor-recipient, sex-matched, n (%)					0.40
M-M	11 (39.3)	5 (26.3)	20 (55.6)	15 (45.5)	
M-F	10 (35.7)	8 (42.1)	9 (25.0)	7 (21.2)	
F-M	3 (10.7)	5 (26.3)	4 (11.1)	8 (24.2)	
F-F	4 (14.3)	1 (5.3)	3 (8.3)	3 (9.1)	
ABO matched, n (%)					0.08
Matched	10 (35.8)	15 (78.9)	17 (47.2)	13 (39.4)	
Major mismatch	9 (32.1)	3 (15.8)	5 (13.9)	11 (33.3)	
Minor mismatch	7 (25.0)	0 (0.0)	9 (25.0)	7 (21.2)	
Bidirectional mismatch	2 (7.1)	1 (5.3)	5 (13.9)	2 (6.1)	
Stem cell source, n (%)					0.16
PB	9 (32.1)	2 (10.5)	14 (38.9)	7 (21.2)	
BM	1 (3.6)	2 (10.5)	0 (0.0)	1 (3.0)	
BM+PB	18 (64.3)	15 (79.0)	22 (61.1)	25 (75.8)	
Disease status, n (%)					0.54
CR1/CR2	25 (89.3)	18 (94.7)	29 (80.6)	28 (84.8)	
>CR2	3 (10.7)	1 (5.3)	7 (19.4)	5 (15.2)	
Conditioning regimen, n (%)					0.67
Bu/Cy	23 (82.1)	18 (94.7)	33 (91.7)	30 (90.9)	
TBI/Cy	4 (14.3)	1 (5.3)	1 (2.8)	2 (6.1)	
Others	1 (3.6)	0 (0.0)	2 (5.6)	1 (3.0)	
Median number of MNC cell, (range), ×10^8^/kg	9.34 (3.70-23.65)	11.02 (3.38-16.57)	11.86 (3.73-19.85)	8.17 (2.97-21.45)	0.09
Median number of CD+34 cell, (range), ×10^6^/kg	3.94 (2.00-8.04)	3.27 (2.02-7.02)	3.95 (1.67-7.88)	3.29 (1.90-11.55)	0.45
HLA matched, n (%)					0.06
5/10	24 (85.7)	14 (73.7)	29 (80.6)	18 (54.5)	
6/10	2 (7.1)	1 (5.3)	6 (16.6)	8 (24.2)	
7/10	2 (7.1)	2 (10.5)	1 (2.8)	5 (15.2)	
≥8/10	0 (0.0)	2 (10.5)	0 (0.0)	2 (6.1)	

Group 1, HLA antibody persistently negative group. Group 2, the *de novo* HLA antibody transiently positive group. Group 3, the *de novo* HLA antibody non-persistently positive group. Group 4, the *de novo* HLA antibody persistently positive group.

AML, acute myeloid leukemia. ALL, acute lymphoblastic leukemia. MDS, myelodysplastic syndrome. CR1, first complete remission. CR2, second complete remission. >CR2, includes third complete remission, part remission, no remission. Bu, busulfan. Cy, cyclophosphamide. TBI, total body irradiation. The HLA allele-matched ≥8/10 group included HLA 9/10 and 10/10 matches. M, male; F, female; BM, bone marrow; PB, peripheral blood.

### 3.2 HLA antibody follow-up results

During the HLA antibody follow-up of 116 patients, 322 samples including 116 pre-transplantation samples, 12 samples at +15 days, 104 samples at 1 months, 74 samples at 3 months, 11 samples at 6 months, and 5 samples at 1 year. In total, 43, 59, 11, and 4 patients were tested once, twice, and three and four times after transplantation, respectively. **Table 2** shows the number of samples and test results of different follow-up time points.

**Table 2:** Number of samples collected at different follow-up times after transplantation and test results.

15 days			1 Month			3 Months			6 Months		
samples	DSA+NDSA	NDSA	samples	DSA+NDSA	NDSA	samples	DSA+NDSA	NDSA	samples	DSA	NDSA
n=12	n=2	n=8	n=104	n=14	n=58	n=74	n=10	n=28	n=11	n=1	n=4
pos, n=10 (83.3%)	DSA: II=2	II=5	pos, n=72 (69.2%)	DSA: II=13; I=1	I+II=24	pos, n=38 (51.4%)	DSA: I=1; II=9	I+II=11	pos, n=5 (45.5%)	DSA: II=1	I+II=2
	NDSA: II=1; I+II=1	I+II=3		NDSA: I+II=8; II=6	I=4; II=30		NDSA: I+II=3; II=7	I=5; II=12		NDSA: II=1	I=1; II=1
neg, n=2 (16.7%)			neg, n=32 (30.8%)			neg, n=36 (48.6%)			neg, n=6 (54.5%)		

neg, negative; pos, positive. I, HLA-I Abs positive; II, HLA-II Abs positive. I+II, HLA-I and II Abs positive.

### 3.3 Analysis of production time, type, and MFI level of *de novo* HLA antibodies

**Figure 1:**
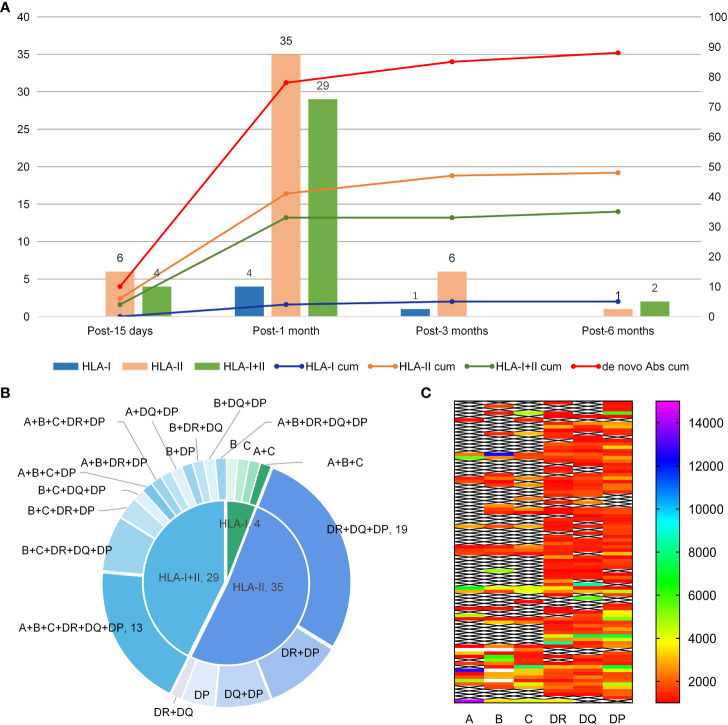
Production time, type, and MFI level of *de novo* HLA antibodies. **(A)** Type of *de novo* HLA antibodies detected at different times after haplo-HSCT. The Y-axis represents the quantity of *de novo* HLA antibodies. The bars correspond to the left Y-axis; the lines correspond to the right Y-axis and represent the cumulated quantity of antibodies; cum, reprensents accumulation. **(B)** Specificity of *de novo* antibodies detected 1 month after transplantation. **(C)** MFI level distribution of *de novo* antibodies in 88 patients.

*De novo* HLA Abs were detected in 88 patients during follow-up and the detection rate was 75.9% (88/116). The median MFI for *de novo* HLA antibodies was 2439 (1033-20162). Antibodies were detected in 10, 68, 7, and 3 patients on 15 days, 1 month, 3 months, and 6 months after transplantation. Of these patients with *de novo* HLA antibodies, five patients were positive for HLA-class I antibodies, 48 were positive for HLA-class II antibodies, and 35 were positive for both HLA-class I and II antibodies. Out of all 40 patients with HLA-I antibodies, the detection rates for A, B, and C antibodies were 20.7% (24/116), 31.0% (36/116), and 28.4% (33/116), respectively. Of the 83 patients with HLA-II antibodies, the detection rates for DR, DQ, and DP antibodies were 59.5% (69/116), 56.0% (65/116), and 66.4% (70/116), respectively, and the quantity of *de novo* antibodies was the highest at 1 month. **Figure 1A** shows the types of *de novo* HLA antibodies produced at different follow-up time points. **Figure 1B** shows the specific types of *de novo* HLA antibodies at 1 month. The MFI level of *de novo* specific antibody types in 88 patients were calculated based on the first time when *de novo* HLA antibodies were detected and 38, 36, 7, and 7 patients had MFI levels of 1,000–2,000, 2,000–5,000, 5,000–8,000, and over 8000, respectively (**Figure 1C**).

### 3.4 Analysis of production time, type, and level of persistent *de novo* HLA antibodies

Out of 33 patients in group 4, *de novo* HLA antibodies were detected in 8, 24, and 1 patient at 15 days, 1 month, and 3 months after transplantation, respectively. The HLA genotyping results of the donor showed that 8 patients had *de novo* DSA + NDSA (**Table 3**, patients 1–8, group 4a) and 25 patients had *de novo* NDSA (**Table 3**, patients 9–23, group 4b). **Table 3** shows the gender, age, and relationship of group 4 patients and their donors, diagnosis of patients, type of *de novo* antibodies, MFI level changes, and patient outcomes.

**Table 3:** Basic clinical information, antibody type and outcomes of patients with persistent *de novo* HLA antibodies after haplo-HSCT (n=33).

Patients	Age (y)	Sex	Diagnosis	Donor	Donor age (y)	HLA Abs type	HLA Abs MFI				Outcome
							15 days	1 M	3 M	MFI change
1	33	M	MDS	Mother	55	A+B+C+DR+DQ+DP	DSA-DR14:1595	DSA-DR14:758	*	↓	Alive
							NDSA-Cw17:3940	NDSA-Cw17:1688		
2	31	M	AML	Father	55	DR+DQ+DP	DSA-DR8:656	/	DSA-DR8:1670	↑	Died
							NDSA-DR7:1310		NDSA-DR16:3400	
3	31	M	AML	Sister	30	DR+DQ+DP	/	DSA-DQ2:1385	DSA-DQ2:1049	↓	Died
								NDSA-DQ6:7363	NDSA-DP19:1721	
4	27	M	ALL	Father	46	DR+DQ+DP	/	DSA-DQ4:1687	DSA-DQ4:1164	↓	Alive
								NDSA-DP1:4343	NDSA-DP1:1069	
5	29	M	ALL	Father	48	A+B+C+DR+DQ+DP	/	DSA-A11:550	DSA-A11:1005	↑	Died
								NDSA-DQ2:1488	NDSA-DQ2:1879	
6	58	F	AML	Daughter	34	A+B+C+DR+DQ+DP	/	DSA-DQ7:1132	DSA-DQ7:1017	↓	Alive
								NDSA-B76:2721	NDSA-B76:1868	
7	33	F	ALL	Father	60	A+B+C+DR+DQ+DP	/	DSA-DR14:1710	DSA-DR14:1142	↓	Died
							NDSA-DQ8:2238	NDSA-DQ8:1608		
8	57	M	Lymphoma	Uncle	39	DR+DQ+DP	/	DSA-DQ9:1562	DSA-DQ9:1376	↓	Died
								NDSA-DP19:2005	NDSA-DP19:1327	
9	37	M	AML	Son	11	DR+DP	/	DR16:1141	DP1:1676	↑	Died
10	23	M	AML	Father	53	DQ	DQ6:5634	DQ6:4539	/	↓	Died
11	22	M	ALL	Father	45	C	/	Cw17:1070	Cw17:1802	↑	Died
12	49	M	AML	Son	25	B+C+DP	/	DP1:1297	Cw17:2202	↑	Alive
13	44	M	MDS	Daughter	20	A+B+DR+DP	/	DP1:1032	DP1:1447	↑	Died
14	52	F	ALL	Daughter	25	DR+DQ+DP	DP1:3697	/	DP19:1666	↓	Alive
15	34	M	AML	Father	56	A+DQ+DP		A24:4720	DP19:1683	↓	Alive
16	52	M	MDS	Daughter	28	DR+DQ+DP	DR7:8137	DR7:11209	DR7:6555	↓	Died
17	56	M	MDS	Daughter	31	A+B+C+DR+DQ+DP	B58:14458	B58:15521	B58:2600	↓	Alive
18	23	F	AML	Father	46	DR+DQ+DP	/	DR14:2020	DP5:1486	↓	Alive
19	20	M	ALL	Father	47	B+C+DR+DQ+DP	/	B45:3322	Cw17:2492	↓	Alive
20	29	F	MDS	Brother	31	DR+DQ+DP	/	*	DQ8:2034	↓	Alive
21	28	M	AML	Father	54	DR+DQ+DP		DQ8:1677	DQ8:1012	↓	Alive
22	48	F	MDS	Daughter	27	A+B+Cw	/	B27:20162	B27:17958	↓	Alive
23	11	F	ALL	Father	37	A+B+Cw+DP	/	A3:3246; DP1:1771	*	↓	Died
24	17	M	AML	Father	40	A+B+C+DR+DQ+DP	/	DP1:1434	DP1:2361	↑	Alive
25	20	M	ALL	Father	48	B+C+DQ+DP	/	B58:5336; DQ4:675	B58:2934; DQ4:1584	↑	Died
26	35	F	AML	Son	12	A+B+C+DR+DQ+DP	/	DR16:1004	DR16:1590	↑	Died
27	48	M	ALL	Daughter	23	B+C+DR+DQ+DP	/	DP1:7051	DP19:1387	↓	Alive
28	43	M	AML	Daughter	16	A+B+C+DR+DQ+DP	/	B58:2015	A11:2926	↑	Died
29	24	M	AML	Father	49	A+B+C+DR+DQ+DP	B45:17082	/	*	↓	Died
30	17	F	AML	Father	38	A+B+C+DR+DQ+DP	DR14:2245	/	DR14:4700	↑	Alive
31	22	M	AML	Father	46	DR+DQ+DP	/	DP1:2101	DQ2:7900	↑	Died
32	40	F	AML	Son	15	DQ+DP	/	DQ8:708; DP19:425	DQ8:1097; DP19:1249	↑	Died
33^※^	58	M	AML	Daughter	28	A+B	/	A24:14276(6 M)	A24:15553(1 Y)	↑	Died

If the patient had multiple types of *de novo* specific antibodies, the specific antibody site with the highest MFI value was analyzed in the table as an example.

* Represents a positive mixed antibody (MIX) test result; no specific test was performed at this follow-up point, and the change in MFI level was determined by the MIX result./represents not detected.

An increase in MFI value above 300 is defined as MFI increasing and is represented by ↑. A decrease in MFI value above 300 is defined as MFI decreasing and is represented by ↓.

※Patient 33 shows the results of NDSA-specific tests at 6 M and 1 year after haplo-HSCT. M, male; F, female.

**Figure 2:**
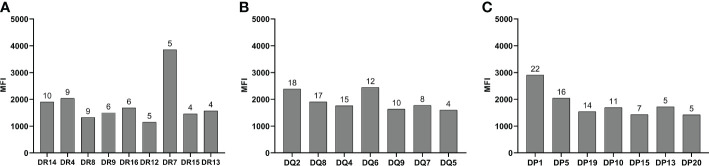
Type of HLA-DR, DQ, and DP specific antibodies. **(A)** DR antibody specificity type. **(B)** DQ antibody specificity type. **(C)** DR antibody specificity type. The numbers on the bars represent the number of patients positive for the specific antibodies.

Of these patients with persistent *de novo* HLA antibodies, three patients were positive for HLA-class I antibodies, 13 were positive for HLA-class II antibodies, and 17 were positive for both HLA-class I and II antibodies. The distribution of the different specific antibody types detected in DR, DQ, and DP antibodies is shown in **Figure 2**.

### 3.5 Therapy and dynamic detection of persistent *de novo* HLA antibodies

Three of the patients with persistent *de novo* HLA antibodies received HLA antibody therapy. The first patient showed *de novo* HLA-II NDSA at 1-month post-transplantation with a maximum MFI value of 1114. This patient developed severe aGvHD at 1.8 months and was treated with plasmapheresis combined with rituximab at 2 months. SAB tests showed NDSA levels at 3 months with a maximum MFI of 1676. This patient had persistent low platelets and died at 14 months due to disease relapse.

In the second patient, HLA-II NDSA was detected at 15 days post-transplantation with a maximum MFI value of 8137. This patient had persistently low platelet counts, and the MFI level increased to 11209 at 1 month. After treatment with plasmapheresis combined with rituximab at 2 months, the platelet count gradually increased and the MFI decreased to 6555 at 3 months. This patient eventually died at 9 months due to multiple organ dysfunction syndrome.

In the third patient, HLA-I+II NDSA was detected at 15 days and 3 months post-transplantation, with the highest MFI values of 2245 and 4700, respectively. The patient developed low platelets and was treated with plasmapheresis combined with rituximab. The MFI decreased to 3821 at 6 months, but platelet count continued to be low. The patient survived to the follow-up endpoint.

## 4 Effects of *de novo* HLA antibody on haplo-HSCT outcomes

### 4.1 Hematopoietic reconstitution

#### 4.1.1 Platelet reconstitution

The median platelet reconstitution time was 15 (10–36) days, 16 (10–98) days, 16 (10–70) days, and 14 (10–154) days in groups 1, 2, 3, and 4, respectively (P = 0.49). The median platelet reconstitution time was 12 (10–21) and 15 (10–154) days in group 4a and group 4b, respectively. There were no statistical differences between the two groups and group 1 (P=0.32).

#### 4.1.2 Incidence of platelet transfusion dependence

Platelet transfusion dependence was observed in 33 patients and total incidence was 28.4% (33/116). The total incidence was 17.9% (5/28), 21.1% (4/19), 27.8% (10/36), and 42.4% (14/33) in groups 1, 2, 3, and 4, respectively (P = 0.16). However, the total incidence of group 4 was significantly higher than that of group 1 (P = 0.04).

#### 4.1.3 Graft rejection

Only 1 out of 116 enrolled patients developed graft rejection. In this patient, neutrophil and platelet counts observed at 28 days after transplantation did not reach the standard for hematopoietic recovery, and short tandem repeat chimerism analysis showed neither the appearance nor complete loss of donor-derived neutrophils. The pre-transplantation and post-transplantation HLA antibody test results of this patient were both negative and grafts were successfully transplanted in other patients.

### 4.2 aGvHD

#### 4.2.1 Incidence of aGvHD in the four groups

**Figure 3:**
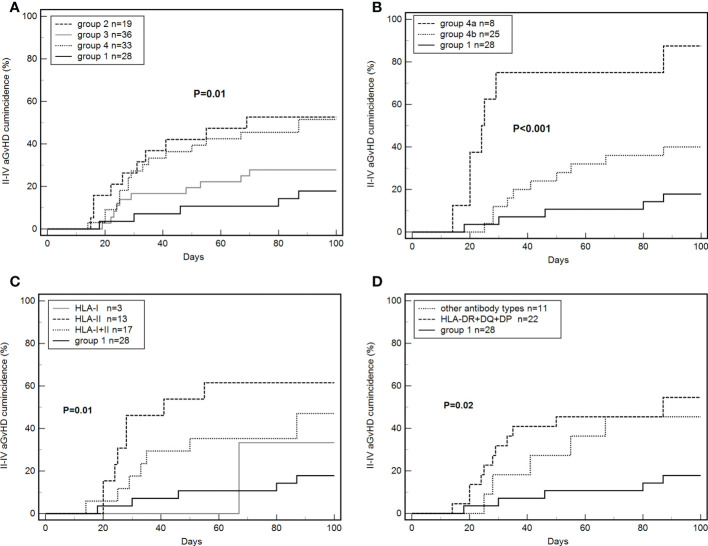
Effects of *de novo* antibodies on grade II–IV aGvHD. **(A)** *De novo* HLA antibodies in four groups. **(B)** Persistent *de novo* antibodies. **(C)** Different *de novo* HLA antibody types. **(D)** *De novo* HLA-DR+DQ+DP antibodies.

The cumulative incidence of grade II–IV aGvHD within 100 days in all enrolled patients was 17.9% (5/28), 52.6% (10/19), 27.8% (10/36), 51.5% (17/33), respectively, in groups 1, 2, 3, and 4 (P = 0.01, **Figure 3A**). The incidence of grade II–IV aGvHD in group 2 was significantly higher than in group 1 (HR: 4.47, 95% CI: 1.52–13.12; P < 0.01), and it was significantly higher in group 4 than in group 1 (HR: 3.30, 95% CI: 1.42–7.68; P = 0.01).

#### 4.2.2 Effects of persistent *de novo* antibodies on aGvHD

##### 4.2.2.1 *De novo* DSA+NDSA and NDSA

The incidence of grade II–IV aGvHD was 87.5% (7/8) and 40.0% (10/25) in groups 4a and 4b, respectively. The differences between groups 4a, 4b, and 1 were significant (P < 0.001, **Figure 3B**). The incidence of grade II–IV aGvHD in group 4a was significantly higher than in group 1 (87.5% vs 17.9%; HR: 60.44, 95% CI: 10.97–332.89; P < 0.001). Group 4b tended to have higher values than group 1 but the difference was not significant (40.0% vs 17.9%, P = 0.07). Group 4a was significantly higher than group 4b (87.5% vs 40.0%, HR: 12.64, 95% CI: 3.02–52.94; P = 0.001).

The cumulative incidence of grade III–IV aGvHD within 100 days in all of the enrolled patients was 17.24% (20/116). The incidence of grade III–IV aGvHD was 62.5% (5/8), 20.0% (5/25), and 7.1% (2/28) in groups 4a, 4b, and 1, respectively. Specifically, the incidence of grade III–IV aGvHD was significantly higher in group 4a than in group 1 (HR: 78.01, 95% CI: 9.84–618.27; P < 0.001) and group 4b (HR: 11.93, 95% CI: 2.2–64.65; P = 0.004).

##### 4.2.2.2 MFI level changes

The 33 patients with persistent *de novo* antibodies were divided into the MFI increasing group (n=14) and MFI decreasing group (n=19) according to the results in **Table 3**. The incidence of grade II–IV aGvHD was 42.9% (6/14) and 57.9% (11/19) in the increasing and decreasing groups, respectively (P = 0.33).

##### 4.2.2.3 HLA antibody type

The cumulative incidence of grade II–IV aGvHD was 33.3% (1/3), 61.5% (8/13), and 47.1% (8/17) in patients with *de novo* HLA-I, HLA-II, and HLA-I+II antibodies, respectively. The three groups had significant differences when compared with group 1 (P=0.01, **Figure 3C**).

A total of 22 patients were positive for HLA-DR+DQ+DP antibodies and 11 patients for other antibodies. The incidence of grade II–IV aGvHD was 54.6% (12/22) and 45.4% (5/11) in the two groups, with significant differences from group 1 (P=0.02, **Figure 3D**).

### 4.3 Relapse

By the end of the follow-up, 16 patients relapsed, and the relapse rate was 13.8%. The cumulative relapse rates of groups 1, 2, 3, and 4 were 10.7% (3/28), 0.0% (0/19), 13.9% (5/36), and 24.2% (8/33), respectively (P=0.11). The relapse rate of group 4 tended to be higher than that of group 1 but the difference was not significant (24.2% vs 10.7%, P = 0.13).

The cumulative relapse rates of groups 4a, 4b, and 1 were 12.5%, 28.0%, 10.7% (P = 0.22). The relapse rate of the MFI increasing group and MFI decreasing group was 35.7% (5/14) and 15.8% (3/19), respectively (P = 0.27).

### 4.4 NRM

The cumulative incidence of NRM was 10.7% (3/28), 26.3% (5/19), 16.7% (6/36), and 30.3% (10/33) in groups 1, 2, 3, and 4, respectively (P = 0.31). The incidence of NRM in group 4 tended to be higher than that in group 1 (P = 0.08).

The incidence of NRM was 50.0% (4/8), 24.0% (6/25), and 10.7% (3/28) in groups 4a, 4b, and 1, respectively (P = 0.04). The incidence of NRM was significantly higher in group 4a than in group 1 (HR: 4.40; 95% CI: 1.22–5.08; P = 0.01), and tended to be higher in group 4b than in group 1 (P = 0.25) and in group 4a than in group 4b (P = 0.11).

### 4.5 DFS and OS

#### 4.5.1 DFS and OS of the four groups

**Figure 4:**
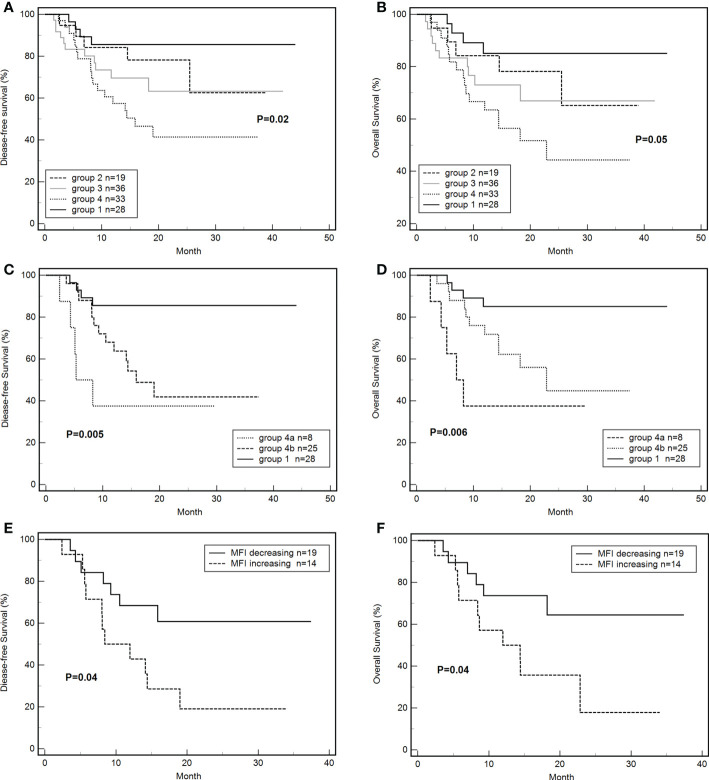
Effects of *de novo* HLA antibody, persistent *de novo* HLA antibody, and changes in MFI on DFS and OS. **(A)** Effects of *de novo* HLA antibodies in four groups on DFS. **(B)** Effects of *de novo* HLA antibodies in four groups on OS. **(C)** Effects of persistent *de novo* antibodies on DFS. **(D)** Effects of persistent *de novo* antibodies on OS. **(E)** Effects of MFI level changes of *de novo* antibodies on DFS. **(F)** Effects of MFI level changes of *de novo* antibodies on OS. Tables.

By the end of the follow-up, 35 out of 116 patients died. The DFS of groups 1, 2, 3, and 4 were 85.7% (24/28), 73.7% (14/19), 69.4% (25/36), 45.5% (15/33), respectively (P = 0.02, **Figure 4A**). The total OS was 69.8%. The OS of groups 1, 2, 3, and 4 were 85.7% (24/28), 73.7% (14/19), 72.2% (26/36), 51.5% (17/33), respectively (P = 0.05, **Figure 4B**). The DFS (HR: 3.54, 95% CI: 1.53-8.17; P < 0.01) and OS (HR: 3.25; 95% CI: 1.35-7.81; P < 0.01) of group 4 were significantly lower than those of group 1. The DFS (P = 0.09) and OS (P = 0.13) of group 3 tended to be lower than those of group 1 but these differences were not significant. The DFS (33.3% vs. 80.7%; HR: 9.59, 95% CI: 4.37-21.06; P < 0.001) and OS (36.4% vs. 83.1%; HR: 10.02, 95% CI: 4.46-22.55; P < 0.001) of platelet-transfusion-dependent patients were significantly lower than those of patients who were not platelet transfusion dependent.

#### 4.5.2 Effects of persistent *de novo* antibodies on DFS and OS

##### 4.5.2.1 *De novo* DSA + NDSA and NDSA

The differences in DFS (37.5% vs 48.0% vs 85.7%, P < 0.01, **Figure 4C**) and OS (37.5% vs 56.0% vs 85.7%, P < 0.01, **Figure 4D**) of groups 4a, 4b, and 1 were significant. The DFS (HR: 15.85, 95% CI: 2.76-91.02; P < 0.01) and OS (HR: 17.58; 95% CI: 2.30-103.06; P < 0.01) of group 4a were significantly lower than those of group 1. The DFS (HR: 3.59, 95% CI: 1.38-9.37; P < 0.01) and OS (HR: 3.11; 95% CI: 1.12-8.62; P = 0.03) of group 4b were significantly lower than those of group 1. The differences of DFS (P =0.27) and OS (P=0.17) between group 4a and 4b were not significant.

##### 4.5.2.2 MFI level changes

The DFS of the MFI increasing group was 21.4% (3/14), which was significantly lower than that of the MFI decreasing group (63.2%,12/19) (HR: 2.77, 95% CI: 1.07-7.22; P = 0.04, **Figure 4E**). Similarly, the OS of the MFI increasing group was 28.6% (3/14), which was significantly lower than that of the MFI decreasing group (68.4%, 13/19) (HR: 2.81, 95% CI: 1.03-7.75; P = 0.04, **Figure 4F**).

### 4.6 Multivariate analysis

Multivariate analysis showed that *de novo* HLA antibody being transiently positive (HR: 5.30; 95% CI: 1.71–16.42, P = 0.01) and persistently positive (HR: 5.67; 95% CI: 2.00–16.08, P < 0.01) were both associated with a higher cumulative incidence of II–IV aGvHD. Donor-recipient HLA compatibility greater than 5/10 was a protective factor for grade II–IV aGvHD (HR: 0.34; 95% CI: 0.14-0.80, P = 0.01). Persistently positive *de novo* HLA antibodies were a risk factor for reduced DFS (HR: 6.57; 95% CI: 2.08–20.70, P < 0.01) and OS (HR: 5.51; 95% CI: 1.73–17.53, P < 0.01) in patients (**Table 4**).

**Table 4:** Multivariate analysis of aGvHD, relapse, DFS and OS after haplo-HSCT in 116 patients with malignant hematological diseases.

Factors		aGvHD		relapse		DFS		OS	
		Multivariate analysis		Multivariate analysis		Multivariate analysis		Multivariate analysis	
	n	HR (95%CI)	p-value	HR (95%CI)	p-value	HR (95%CI)	p-value	HR (95%CI)	p-value
HLA Abs									
Group 1	28	1		1		1		1	
Group 2	19	5.30(1.71-16.42)	0.01	/		1.68(0.43-6.61)	0.46	1.87(0.47-7.42)	0.38
Group 3	36	1.84(0.61-5.56)	0.28	1.60(0.38-6.71)	0.52	3.30(0.99-11.03)	0.05	2.95(0.87-10.00)	0.08
Group 4	33	5.67 (2.00-16.08)	0.00	2.65(0.70-10.00)	0.15	6.57(2.08-20.70)	0.00	5.51(1.73-17.53)	0.00
Patient age	116	1.01(0.99-1.04)	0.35	0.98(0.96-1.04)	0.89	1.01(0.99-1.04)	0.40	1.02(0.99-1.04)	0.20
Sex									
Male	74	1	0.96	1	0.44	1	0.96	1	0.87
Female	42	0.98 (0.42-2.27)		0.64(0.21-1.98)		0.98(0.44-2.20)		0.93(0.40-2.18)	
Diease									
AML	45	1		1		1		1	
ALL	43	1.51(0.53-2.49)	0.72	0.84(0.30-2.32)	0.74	1.27(0.58-2.81)	0.55	1.13(0.4-2.58)	0.78
MDS	20	1.59(0.64-3.95)	0.32	/		0.53(0.17-1.65)	0.27	0.56(0.18-1.73)	0.31
Lymphoma	8	3.23(0.94-11.07)	0.06	0.77(0.09-6.17)	0.81	2.35(0.67-8.27)	0.19	1.48 (0.37-5.87)	0.58
Disease status									
CR1 or CR2	100	1	0.55	1	0.88	1	0.55	1	0.44
more than CR2	16	1.30(0.55-3.08)		0.89(0.20-3.96)		1.34(0.52-3.50)		1.46(0.56-3.79)	
Sex matched									
matched	62	1	0.37	1	0.76	1	0.30	1	0.21
mismatched	54	0.71(0.33-1.51)		0.86(0.32-2.30)		1.46(0.72-2.94)		1.59(0.77-3.30)	
ABO matched									
matched	56	1	0.32	1	0.92	1	0.69	1	0.99
mismatched	60	0.70(0.34-1.42)		1.05(0.39-2.82)		0.86(0.39-1.86)		1.00(0.46-2.21)	
HLA matched									
5/10 matched	83	1	0.01	1	0.33	1	0.15	1	0.11
>5/10 matched	33	0.34(0.14-0.80)		0.53(0.15-1.88)		0.52(0.22-1.26)		0.48(0.19-1.19)	
Number of CD34^+^ cells									
≤ 4×10^6^/kg	69	1	0.33	1	0.10	1	0.99	1	0.63
>4×10^6^/kg	47	1.39(0.71-2.72)		0.35 (0.09-1.22)		1.00(0.48-2.07)		1.20(0.57-2.34)	
HLA Abs									
Group 1	28	1.0		1.0		1.0		1.0	
Group 2	19	5.22(1.69-16.11)	0.01	/		1.61(0.40-6.40)	0.50	1.80(0.457.24)	0.41
Group 3	36	1.78(0.59-5.32)	0.31	1.78(0.37-8.49)	0.47	3.35(1.00-11.04)	0.05	3.07(0.92-10.25)	0.07
Group 4	33	5.64 (1.99-16.03)	0.00	4.40(0.88-21.95)	0.07	6.53(2.07-20.54)	0.00	5.64(1.76-18.09)	0.00
Patient age	116								
≤35 years	69	1.0	0.58	1.0	0.36	1.0	0.09	1.0	0.03
>35years	47	1.20(0.64-2.25)		1.68(0.55-5.11)		1.84(0.92-3.71)		2.28(1.09-4.75)	
Sex									
Male	74	1.0	0.91	1.0	0.77	1.0	0.93	1.0	0.97
Female	42	0.95 (0.41-2.12)		0.81(0.20-3.29)		1.04(0.46-2.34)		0.99(0.42-2.30)	
Diease									
AML	45	1.0		1.0		1.0		1.0	
ALL	43	1.11(0.52-2.38)	0.79	1.39(0.42-4.61)	0.59	1.37(0.62-3.02)	0.44	1.23(0.54-2.80)	0.63
MDS	20	1.58(0.64-3.91)	0.32	/		0.56(0.18-1.73)	0.31	0.58(0.19-1.84)	0.37
Lymphoma	8	3.28(0.96-11.25)	0.06	0.60(0.06-5.94)	0.66	2.20(0.63-7.69)	0.22	1.44 (0.37-5.66)	0.60
Disease status									
CR1 or CR2	100	1.0	0.61	1.0	0.64	1.0	0.22	1.0	0.22
more than CR2	16	1.12(0.73-1.69)		1.56(0.25-9.88)		1.32(0.85-2.05)		1.33(0.85-2.08)	
Sex matched									
matched	62	1.0	0.44	1.0	0.52	1.0	0.26	1.0	0.17
mismatched	54	0.74(0.35-1.58)		1.44(0.47-4.42)		1.49(0.75-2.95)		1.66(0.81-3.41)	
ABO matched									
matched	56	1.0	0.36	1.0	0.86	1.0	0.65	1.0	0.96
mismatched	60	0.72(0.36-1.45)		0.90(0.28-2.93)		0.83(0.38-1.81)		0.98(0.44-2.15)	
HLA matched									
5/10 matched	83	1.0	0.01	1.0	0.32	1.0	0.14	1.0	0.10
>5/10 matched	33	0.34(0.14-0.80)		0.51(0.13-1.96)		0.51(0.21-1.25)		0.45(0.18-1.16)	
Number of CD34^+^ cells									
≤ 4×10^6^/kg	69	1.0	0.33	1.0	0.09	1.0	0.98	1.0	0.55
>4×10^6^/kg	47	1.40(0.72-2.71)		0.27(0.06-1.22)		1.01(0.49-2.09)		1.26(0.60-2.63)	

## 5 Discussion

This study showed that post-transplantation dynamic testing of patients without preexisting HLA antibodies will detect *de novo* HLA antibodies after HSCT. In this study, we employed HLA antibody specificity testing to dynamically detect *de novo* HLA antibodies and found that the proportion of patients with *de novo* HLA antibodies was 75.9% and the proportion of patients with persistent *de novo* antibodies was 28.4%. *De novo* HLA antibodies could be detected at 15 days and 1 month after transplantation and the quantity and number of types of *de novo* antibodies was the highest at 1 month. These persistent *de novo* antibodies increased the incidence of post-transplantation aGvHD and severely affected patient survival. Our study results showed that HLA-II antibodies were the main *de novo* HLA antibody, which is consistent with the results of some organ transplantation studies (18–20). This finding is also consistent with our previous study on post-kidney transplantation *de novo* HLA antibodies, showing that post-transplantation *de novo* DSA antibodies were mainly HLA-II antibodies (21). Previous studies in organ transplantation (19, 20, 22, 23) and our study have shown that *de novo* HLA antibodies will affect graft rejection and survival. Therefore, HLA antibodies should be monitored after transplantation, and we recommend that 1-month post-transplantation is the best test time point. Previous studies also showed that HLA antibodies are associated with platelet transfusion refractoriness (24, 25). HLA antibody testing should be carried out when thrombocytopenia, transfusion dependence, or transfusion refractoriness occurs.

A potential explanation why post-HSCT HLA antibodies could contribute to GVHD may be that HLA is expressed on the surface of endothelial cells. In the setting of haplo-identical transplantation, due to donor–recipient HLA mismatch, specific T cells differentiate into effector cells that promote B cells into plasma B cells, which produce HLA antibodies. HLA antibodies crosslinking of HLA molecules on endothelial cells stimulates intracellular signaling, including FAK/Src, ERK, PIK, and mTORC1. HLA molecules trigger these signaling pathways by physically associating with integrin β4 or TLR4, thereby leading to activation of endothelial cells and recruitment of leukocytes, which manifests as transplant vasculopathy (26, 27).

The main reason why *de novo* HLA antibodies are HLA-II antibodies may be that HLA-II molecules can directly recognize foreign antigens and genes encoding the α and β chains of HLA-II antigen are polymorphic (28) and are associated with the intensity of HLA-II antigens. These results suggest that we not only need to carry out HLA antibody specificity testing and HLA high-resolution typing, but donors also need to undergo HLA allele analysis to determine the type of *de novo* antibody specificity and DSA.

In this study, single-antigen beads were used for dynamic testing of HLA antibodies. Single-antigen bead reagents have high antigen density and stronger antibody binding capacity. Additionally, they not only can detect HLA antibody specificity in patients and the type of corresponding HLA allele, thereby identifying DSA, but can also dynamically analyze antigen–antibody interaction strength as measured by MFI level. Thus, they can improve identification of antibody reactivity and specificity, and are widely used in organ transplantation and HSCT (29). The value of dynamic testing of HLA antibody is not limited to discovery of *de novo* HLA antibodies; the types of antibodies and changes in MFI level can also be discovered. This study also demonstrated that *de novo* HLA antibodies and their changes can affect the outcomes of patients who underwent haplo-HSCT. Therefore, dynamic testing of HLA antibodies is extremely important in haplo-HSCT.

There are some potential limitations and unanswered questions in this study. Considering that this research retrospectively analyzed HLA antibodies of patients after transplantation, the uncertainly of sample collection at different time points might have led to the low cumulative incidence of DSA positivity. Our subsequent study based on a prospective design and a larger sample size may compensate for the shortage and allow to explore the potential mechanism. In addition, some studies have focused on donor versus recipient antibodies (RSA). Taniguchi et al. (30) showed that donor-derived HLA antibodies may be present in patients, and Delbos et al. (31) suggested that RSA may be a risk factor for the development of GvHD. RSA testing can be performed on female or older donors if necessary.

In summary, all patients in this study underwent pre-transplantation and post-transplantation HLA antibody dynamic testing using single-antigen bead reagents. We found that patients who were negative for pre-existing HLA antibodies could produce *de novo* HLA antibodies after transplantation, and *de novo* antibodies were mostly detected 1 month after transplantation. Persistent *de novo* HLA antibodies affected the occurrence of aGvHD and long-term survival. Therefore, specific method must be used to detect HLA antibodies after haplo-HSCT in patients who are negative for HLA antibodies before transplantation. We recommend that testing should be carried out 1 month after transplantation. In addition, HLA antibody testing should also be carried out when thrombocytopenia, transfusion dependence, or rejection occurs in the patient.

## Data availability statement

The raw data supporting the conclusions of this article will be made available by the authors, without undue reservation.

## Ethics statement

The studies involving human participants were reviewed and approved by the Institutional Review Board at First Affiliated Hospital of Soochow University. Written informed consent was obtained from the participants and minors’ legal guardian for participation in this study and for the publication of any potentially identifiable images or data included in this article.

## Author contributions

JH contributed to the design of the project and statistical analysis plan, oversaw drafting of the manuscript and provided feedback on the manuscript; LW participated in recruitment of patients and collection of data, analyzed the clinical data and interpretation, and draft the manuscript; KJ participated in recruitment of patients and collection of data; YL and WZ participated in clinical and experimental data analysis; LC, XY and XB participated in the detection and interpretation of HLA antibodies data; XW provided feedback on the manuscript. All authors contributed to the article and approved the submitted version.

## Funding

This work was supported by the National Natural Science Foundation of China (Grant No. 82070180) and Jiangsu Provincial Key Research and Development Program (BE2019656).

## Acknowledgments

The authors thank the Sample Bank of the First Affiliated Hospital of Soochow University, for assistance with the sample retrieval and the Department of Hematology for providing clinical data.

## Conflict of interest

The authors declare that the research was conducted in the absence of any commercial or financial relationships that could be construed as a potential conflict of interest.

## Publisher’s note

All claims expressed in this article are solely those of the authors and do not necessarily represent those of their affiliated organizations, or those of the publisher, the editors and the reviewers. Any product that may be evaluated in this article, or claim that may be made by its manufacturer, is not guaranteed or endorsed by the publisher.
